# Vanadyl Phthalocyanine as a Low-Temperature/Low-Pressure Catalyst for the Conversion of Fructose to Methyl Levulinate

**DOI:** 10.3390/molecules30092065

**Published:** 2025-05-06

**Authors:** Juan Luna, Mataz Alcoutlabi, Elizabeth Fletes, Helia Morales, Jason G. Parsons

**Affiliations:** 1 School of Integrative Biological and Chemical Sciences, University of Texas Rio Grande Valley, 1 West University Blvd, Brownsville, TX 78521, USA; juan.r.luna01@utrgv.edu; 2 Department of Mechanical Engineering, University of Texas Rio Grande Valley, 1201 W University Drive, Edinburg, TX 78539, USA; mataz.alcoutlabi@utrgv.edu (M.A.); elizabeth.fletes01@utrgv.edu (E.F.); 3 School of Earth Environmental and Marine Sciences, University of Texas Rio Grande Valley, 1 W University Blvd, Brownsville, TX 78521, USA

**Keywords:** fructose, methyl levulinate, vanadyl phthalocyanine, heterogenous catalysis, kinetics

## Abstract

In this study, a vanadyl phthalocyanine was synthesized and characterized using XRD, FTIR, and XPS, confirming the successful metalation of the phthalocyanine ring. XRD analysis showed the vanadyl phthalocyanine crystallized in the P-1 crystal lattice, with unit cell parameters a = 12.058 Å, b = 12.598 Å, and c = 8.719 Å, and the lattice angels were 96.203°, 94.941°, and 68.204°. FTIR spectroscopy supported the metalation by the disappearance of the N-H stretch of the non-metalated phthalocyanine. The vanadyl phthalocyanine was tested as a heterogenous catalyst for the conversion of fructose into methyl levulinate in H_2_SO_4_–methanol and HCl–methanol systems. The H_2_SO_4_–methanol reaction system catalyzed with the vanadyl phthalocyanine, and a zeroth-order rate constant of 1.10 × 10^−6^ M/s was observed, which was 1.74 times faster than sulfuric acid alone. The HCl–methanol system showed a zeroth-order of reaction with a rate constant of 2.33 × 10^−6^ M/s, which was 1.3 times faster than the HCl–methanol alone. While the HCl–methanol system showed a faster reaction rate, product distribution favored methyl levulinate formation in the H_2_SO_4_–methanol system. The main products identified were methyl levulinate and hepta-2,4-dienoic acid methyl ester, with a minor amount of hydroxymethylfurfural formed. These results suggest that vanadyl phthalocyanine can be effectively used as a catalyst to increase the rate of fructose conversion to methyl levulinate in either H_2_SO_4_ or HCl–methanol.

## 1. Introduction

As the world moves further away from fossil fuels and petroleum-based products, there is a global demand for alternative sources that are sustainable, cost-effective, and widely accessible. This shift has led to extensive research into novel methods for the synthesis and extraction of critical compounds. The development of synthetic building blocks has become a promising strategy for producing energy-relevant chemicals, polymers, pharmaceuticals, food additives, and other industrially significant compounds [1,2,3]. Catalysis can provide an important option for the synthesis or conversion of starting materials for various chemicals [4,5]. Sugars have emerged as promising starting materials for the development of synthetic fuel precursors [6,7]. Hexose sugars derived from cellulose and other forms of biomass can be converted into valuable compounds such as levulinic acid, which holds significant potential for various applications. Similarly, fructose has been demonstrated to convert into levulinic acid or methyl levulinate [8,9]. The first synthesis of levulinic acid was achieved by the chemist Mulder through the heating of sucrose in the presence of hydrochloric acid [10]. Levulinic acid and its alkyl-substituted alternatives have shown promise as precursors for biofuel synthesis and as additives in petroleum-based products [11,12,13,14]. Furthermore, levulinates are particularly attractive due to their reactivity, which arises from the presence of functional groups such as carboxylic acid, ester, and ketone. Levulinates can be used as precursors in a wide range of reactions, including Fischer esterification, condensations, and addition reactions [13,14,15,16]. As a result, levulinic acid and its derivatives are used in a broad range of applications, including pharmaceuticals, preservatives, and polymers, as well as in the synthesis of γ-valerolactone. γ-valerolactone is a key intermediate in the production of polymers, herbicides, pharmaceutical compounds, anti-freeze agents, plasticizers, solvents, flavoring agents, fragrance compounds, and resins [11,15,16].

The synthesis of levulinic acid and its derivatives from sugars has been reported since the 1870s, with early works by Grote and Tollens confirming its formation through the acid-catalyzed degradation of simple sugars [17,18]. However, conventional synthetic methods often rely on non-sustainable processes and present several drawbacks, including multi-step procedures, extensive purification, high costs, low yields, and the need to operate at high temperatures and pressures [12,14,16]. As a result, there is a growing interest in developing catalytic processes that are both efficient and sustainable for the synthesis of levulinic acid. In recent years, numerous strategies have been explored, incorporating diverse catalyst systems, alternative solvent media, one-pot reactions, and environmentally friendly technologies [12,14,18,19,20,21]. These advances focus on making the production of levulinates more viable at an industrial scale by reducing costs, improving availability, and expanding their potential for research and commercial applications. 

In recent years, a variety of strategies have been developed to improve the production of methyl levulinate. Szabó et al. reported the microwave-assisted, base-catalyzed alkylation of levulinic acid as an efficient synthetic approach [22]. Among the inorganic bases investigated, potassium phosphate was identified as the most effective catalyst, offering advantages in terms of efficiency and reusability. Fuchineco et al. synthesized two distinct zirconium-based metal–organic frameworks (MOFs), one constructed with terephthalic acid ligands and the other with aminoterephthalic acid ligands, demonstrating the efficiency and selectivity of these materials for levulinic acid esterification [23]. The best catalytic performance was observed with the MOF based on aminoterephthalic acid, achieving a yield of 67.8% and representing the most promising alternative. Aho et al. [23] investigated metal-modified Beta zeolites as catalysts for the conversion of glucose to methyl levulinate, with copper modification significantly enhancing catalytic selectivity, achieving 92.6% conversion and a methyl levulinate yield of approximately 89% under optimized conditions. Wang et al. introduced molecular oxygen into the catalytic transformation of 5-hydroxymethylfurfural, successfully promoting methyl levulinate formation while simultaneously suppressing humin generation, thereby improving both product yield and catalyst recyclability [24].

Phthalocyanines have been successfully used as catalysts due to their exceptional structural stability and electronic versatility. Phthalocyanines are planar macrocyclic structures forming an 18 π-electron aromatic system. This conjugation allows them to act as efficient transfer agents, which is further enhanced while metal-substituted. Since 1938, metal-substituted phthalocyanines complexes have been widely used in heterogeneous catalysis across a range of reactions, including the oxidation of aromatic hydrocarbons, alcohols, aldehydes, alkenes, ketones, and sulfur-containing compounds, as well as in dehydrogenation, decarboxylation, isomerization, polymerization, reduction of organic compounds and electrochemical processes [25,26,27,28,29]. Their stability in extreme conditions, such as strong acid media and high temperatures, makes them particularly suitable for demanding chemical processes. Notably, Soroking et al. [30] demonstrated that iron and manganese tetrasulfophthalocyanines catalyze the degradation of halogenated phenols, leading to ring-cleaved products. Tetrasulfophthalocyanine substituted with iron, manganese, cobalt, and/or nickel in conjunction with hydrogen peroxide showed phenol oxidation. Similarly, Kruid et al. [31] reported that iron and manganese phthalocyanines effectively removed endocrine-disrupting compounds, including bisphenol A, 17β-estradiol, estrone, and coumestrol through H_2_O_2_ mediated oxidation in aqueous systems.

In the present study, vanadyl phthalocyanine (VOPC) was synthesized, characterized, and evaluated for its ability to catalyze the conversion of fructose to methyl levulinate in an acidic methanol solution. The synthesis of the VOPC was confirmed using X-ray diffraction (XRD), X-ray photoelectron spectroscopy (XPS), and Fourier-transform infrared spectroscopy (FTIR). The H_2_PC was found to crystallize in the monoclinic C2/n crystal structure, whereas the synthesized VOPC was found to crystallize in the triclinic P-1 space group. The FTIR spectrum of the metalated compound exhibited the absence of the N-H stretching vibration, confirming the successful metal insertion into the phthalocyanine ring. Reaction progress was monitored using electron ionization gas chromatography–mass spectrometry (GC-MS), comparing acidified methanol alone as the control with VOPC in acidified methanol as the catalytic system. VOPC-catalyzed reactions proceeded at higher rates compared to the control reactions. The GC-MS data revealed two major products were formed in the reaction mixture: hepta-2,4-dienoic acid methyl ester and methyl levulinate. In addition, a minor amount of hydroxymethylfurfural was also detected.

## 2. Experimental/Methodology

### 2.1. Material Synthesis

The synthesis of the vanadium-substituted phthalocyanine was achieved following a modified version of the method by Weber and Busch [32]. Briefly, 0.081 mol of phthalic anhydride, 0.045 mol of ammonium chloride, 0.485 mol of urea, 0.0003 mol of ammonium molybdate (Mo was used as a catalyst In this process; Mo acts as a catalyst for the formation of the 1,3-diiminoisoindoline intermediate species), and 0.024 mol of vanadium (III) chloride were thoroughly mixed and ground to a fine powder using a mortar and pestle. The powdered mixture was added to 40 mL of nitrobenzene in a reaction flask, which was then degassed and continuously purged with nitrogen to maintain an inert atmosphere. The reaction mixture was heated at 180 °C with constant stirring for six hours under these conditions. The reaction mixture was cooled to room temperature, and a bluish precipitate was collected using vacuum filtration. The solid was washed with methanol until the odor of nitrobenzene was no longer detectable. The metal-free phthalocyanine, H_2_PC, was synthesized using the same procedure, excluding vanadium (III) chloride from the reaction mixture.

### 2.2. Material Characterization

The synthesized compounds were dried and finely ground to be characterized using FTIR, XRD, and XPS. The synthesized H_2_PC and the corresponding VOPC were characterized using a PerkinElmer Frontier FTIR spectrometer (PerkinElmer, Shelton, CT, USA). Spectra were collected in absorbance mode (A units) over a range of from 4000 to 650 cm^−1^ at a resolution of 2 cm^−1^. The FTIR was equipped with a KBr beam splitter, a universal attenuated total reflectance (UATR) accessory consisting of a zinc selenide crystal, and a lanthanum tantalate detector. A pressure of 90 N was applied to ensure consistent contact with the ATR crystal for all the samples. Powder XRD patterns of the H_2_PC and the VOPC were collected using a Bruker D2 Phaser diffractometer (Bruker Corporation, Billerica, MA, USA) equipped with a cobalt X-ray source (Co K_α_ = 1.789 Å) and an iron filter. The data were collected over a 2θ range of 5° to 50° and a counting time of 2 s per step. Structural refinement was carried out using the Le Bail fitting procedure in the FullProf Suite software (Version 5.10), based on crystallographic data reported in the literature [33,34,35,36,37,38]. XPS spectra were acquired using a Thermo Scientific K-alpha Photoelectron spectrometer (Thermos Fisher Scientific, Waltham, MA, USA) with a monochromatic Al source (k_α_ = 1486.7 eV). Scans were performed using a spot size of 400 μm and an energy step size of 0.1 eV. The data were processed using the Fityk software [39].

### 2.3. Reaction Studies

Catalyzed reactions of fructose were performed in acidified methanol under reflux and a nitrogen atmosphere. A mixture of 1.7 g of d-fructose (Sigma-Aldrich, Inc., St. Louis, MO, USA) and 0.58 g of VOPC, corresponding to a 1:9 mmol ratio of catalyst to d-fructose, was dissolved in 50 mL of methanol, and 1 mL of acid (either H_2_SO_4_ or HCl) was added. Prior to heating, the reaction mixtures were degassed through three vacuum–nitrogen cycles. The reaction was considered to be started when the solution reached the boiling point of the methanol and was monitored over a total of 270 min. After the first 30 min, samples were collected at one-hour intervals for four additional hours. At each time point, three independent samples were collected and analyzed using GC-MS. Control reactions were performed using the same conditions, excluding the catalyst and consisting of fructose in acidified methanol.

Reaction products were analyzed using a PerkinElmer TurboMass Gold mass spectrometer (PerkinElmer, Shelton, CT, USA) to determine compound identity and relative concentration. Product identification was based on a comparison with the NIST GC-MS spectral library (Gaithersburg, MD, USA). The GC-MS operating conditions were as follows: splitless injections of 1 μL volume, injector temperature of 260 °C, helium carrier gas at a pressure of 10 psi, and a diphenyl column. The oven temperature was ramped up from 50 to 250 °C at a rate of 25 °C/min and the final temperature was held for 2 min. The mass spectrometer was operated in electron ionization (EI) mode with the ion source maintained at 250 °C.

## 3. Results and Discussion

**Figure 1:**
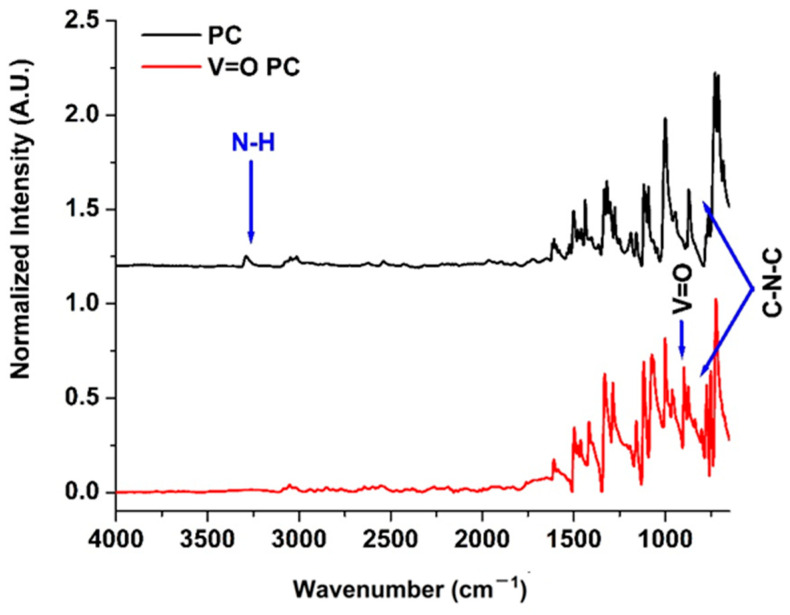
ATR-FTIR spectrum for phthalocyanine (TOP) and V=O phthalocyanine bottom.

Figure 1 shows the FTIR spectra, and Table 1 presents the peak assignments of both the H_2_PC and the VOPC. The most prominent spectral difference between the two compounds is the disappearance of the N–H stretching vibration at 3582 cm^−1^ in the H_2_PC, which is absent in the VOPC. This change confirms the coordination of the vanadium center to the nitrogen atoms, indicating the successful removal of the inner protons and metal insertion into the macrocycle [40,41,42,43]. A shift in the C-N stretching of the isoindole ring at 1299 cm^−1^ in the H_2_PC to 1284 cm^−1^ in the vanadyl-substituted compound further supports coordination between the vanadium and the isoindole nitrogen atoms [40,42]. The appearance of the new peak at 953 cm^−1^ in the VOPC corresponds to the V=O stretching vibration, confirming the presence of the vanadyl moiety [40,42]. Additional changes include a shift in the benzene ring deformation mode, coupled with aza (C–N) deformation, from 944 cm^−1^ in the metal-free compound to 964 cm^−1^ after metalation [40,44]. The characteristic C-N-C ring breathing mode at 839 cm^−1^, present in the metal-free spectrum, is absent in the VOPC [45]. Moreover, a band at 801 cm^−1^, attributed to isoindole stretching coupled with N-V-N asymmetric stretching, appears in the VOPC [45,46].

**Figure 2:**
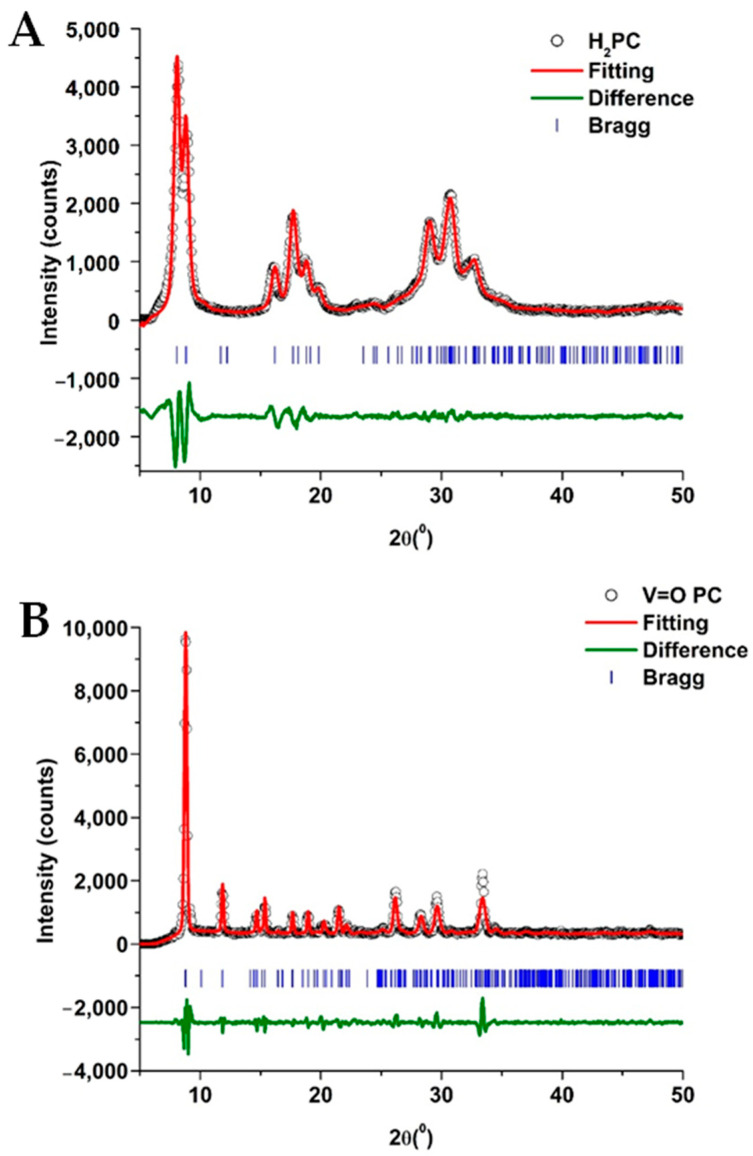
XRD and Le Bail fitting of the H_2_PC (**A**) and the VOPC (**B**) as synthesized.

Figure 2 shows the XRD patterns and the Le Bail fittings for the H_2_PC and the VOPC. The results of the fitting and the determined lattice parameters are summarized in Table 2. The non-metalated phthalocyanine was determined to crystallize in the monoclinic C2/n space group, with unit cell parameters a = 25.755 Å, b = 3.773 Å, and c = 23.398 Å, which are in agreement with the values found in the literature [35,39]. The metal-substituted phthalocyanine crystallized in the triclinic P-1 space group with unit cell parameters a = 12.058 Å, b = 12.598 Å, and c = 8.719 Å, which is also consistent with the literature [37,38]. The Le Bail refinements resulted in reduced χ^2^ values of 2.07 and 1.67 for the H2PC and VOPC, respectively. Reduced χ^2^ values below 5 indicate good agreement between the fitting and the data [40,45,50]. The fitting figures show a low residual in the difference between the fitting and the data, further supporting the accuracy of the refinements.

**Figure 3:**
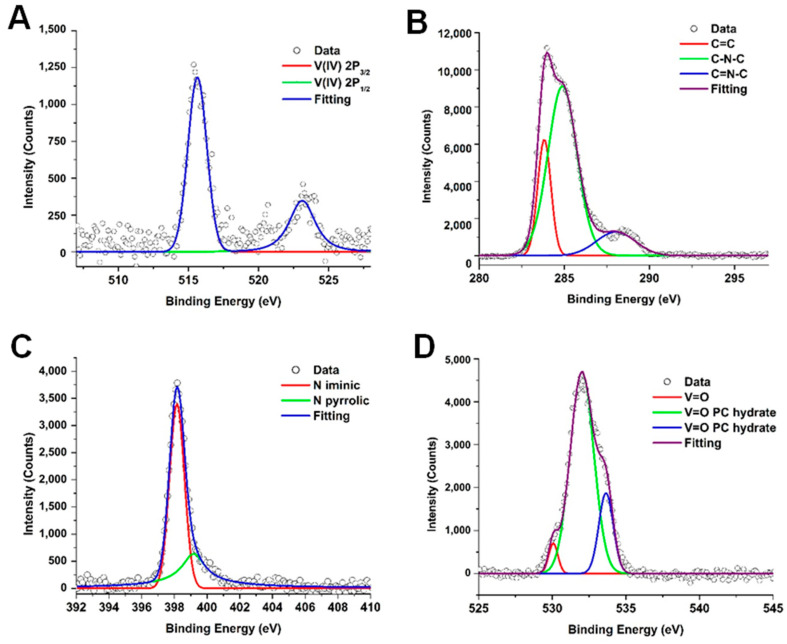
X-ray photoemission spectrum for the synthesized vanadyl phthalocyanine for V 2P (**A**), C 1S (**B**), N 1S (**C**), and O 1S (**D**) regions.

Figure 3 presents the XPS spectra of the synthesized vanadyl phthalocyanine, VOPC. Figure 3A shows the V 2p region, which includes the V 2p_3/2_ and the V 2p_½_ spectral regions. The V 2p_3/2_ region was deconvolved into one peak located at approximately 517 eV, which indicates the presence of a V^4+^ metal ion. The V 2p_1/2_ region had one peak centered around 523 eV, which again is consistent with the presence of a V^4+^. The binding energies of the V 2p peaks are consistent with the literature for vanadyl bound into a phthalocyanine ring system [40]. The C 1s region, shown in Figure 3B, was deconvolved into three peaks located at 283.8, 284.4, and 285.4 eV. These binding energies are attributed to C-C, C-N, and C=N bonding environments, respectively, and are characteristic of metal-substituted phthalocyanines [40]. Figure 3C shows the spectrum of the spectrum of the N 1s region, which exhibited one broad peak that was deconvolved into two components corresponding to pyridinic and pyrrolic nitrogen species, located at 397.8 and 399.5 eV, respectively. The pyrrolic peak is slightly higher than normal, which indicates binding to a metal ion and is consistent with the metal phthalocyanine [40]. The O 1S spectrum is shown in Figure 3D shows, which contained one peak that was deconvolved into three peaks. The peak located at 531.4 eV was identified as the V=O peak [40,45], while peaks at 530.5 eV and 533.2 eV correspond to the surface-bound OH and adsorbed water molecules [40].

**Figure 4:**
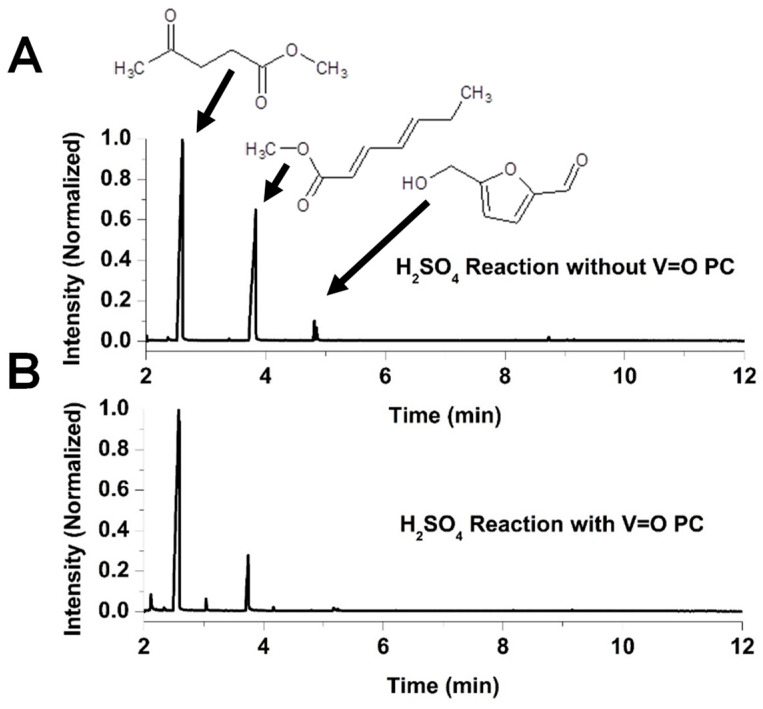
Chromatogram for the reaction of H_2_SO_4_ with fructose in methanol (**A**) and chromatogram for the reaction of H_2_SO_4_ with fructose in methanol with VOPC (**B**).

**Figure 5:**
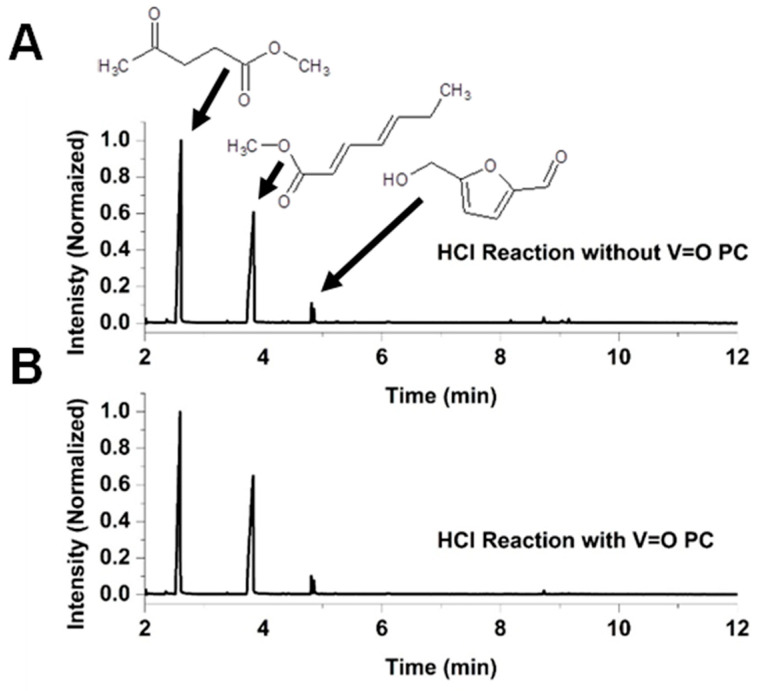
Chromatogram for the reaction of HCl with fructose in methanol (**A**) and chromatogram for the reaction of HCl with fructose in methanol with VOPC (**B**).

Figure 4 presents the chromatograms of the reaction mixtures obtained after 270 min of reaction using VOPC as a catalyst, as well as the corresponding uncatalyzed control reaction in a methanol system acidified with H_2_SO_4_. Fructose was not detected in either reaction mixture. Figure 5 shows chromatograms for the reactions performed in the HCl–methanol system after the same reaction time of 270 min of reaction. Both acidic systems yielded two major reaction products, methyl levulinate and hepta-2,4-dienoic acid methyl ester, along with a minor product, hydroxymethylfurfural (HMF). The chromatograms indicate that the vanadyl-catalyzed reaction in the H_2_SO_4_ system produced significantly higher amounts of methyl levulinate compared to the uncatalyzed reaction, with virtually no detectable HMF, suggesting that vanadyl phthalocyanine effectively drives the reaction towards the formation of the methyl levulinate. Conversely, in the HCl system, the product ratio showed minimal differences between catalyzed and uncatalyzed reactions, indicating that the presence of the VOPC had a limited effect on product selectivity in this acid environment.

**Figure 6:**
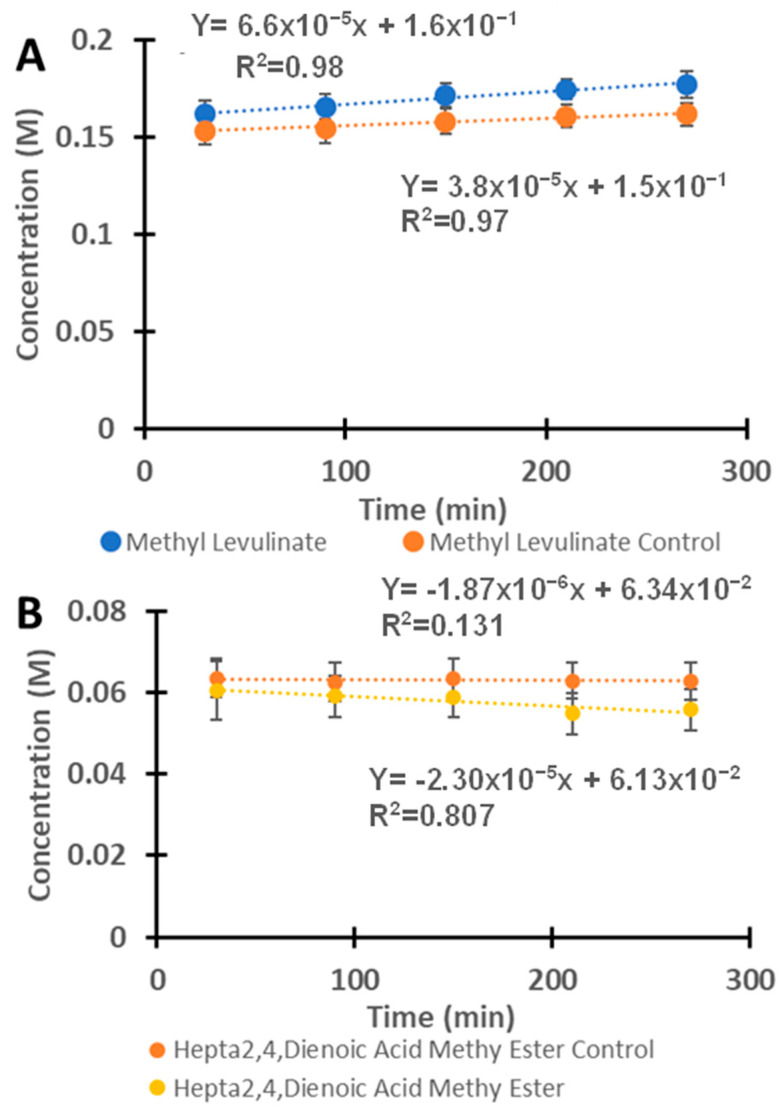
(**A**) The kinetics of the reaction between fructose and H_2_SO_4_ with and without the presence of the vanadyl phthalocyanine for the formation of methyl levulinate. (**B**) The reaction of fructose with H_2_SO_4_ in the presence of vanadyl phthalocyanine as a catalyst for the formation of hepta-2,4-dienoic acid methyl ester.

Figure 6A shows the kinetics of methyl levulinate production using H_2_SO_4_ with and without VOPC. The reaction was observed to follow a zero-order or pseudo-zero-order kinetics rate for methyl levulinate synthesis, with a rate constant of 6.6 × 10^−5^ M/min (or 1.1 × 10^−6^ M/s) for the catalyzed reaction and 3.8 × 10^−5^ M/min (or 6.33 × 10^−7^ M/s) for the uncatalyzed reactions. The presence of VOPC approximately doubled the rate of methyl levulinate production. The hepta-2,4-dienoic acid methyl ester appeared as a product, initially forming and followed by a slight decrease, as indicated by the negative slope of the reaction in Figure 6B. Its concentration, however, remained relatively stable over time, suggesting that it might be an intermediate species in the formation of methyl levulinate in the H_2_SO_4_–methanol system.

**Figure 7:**
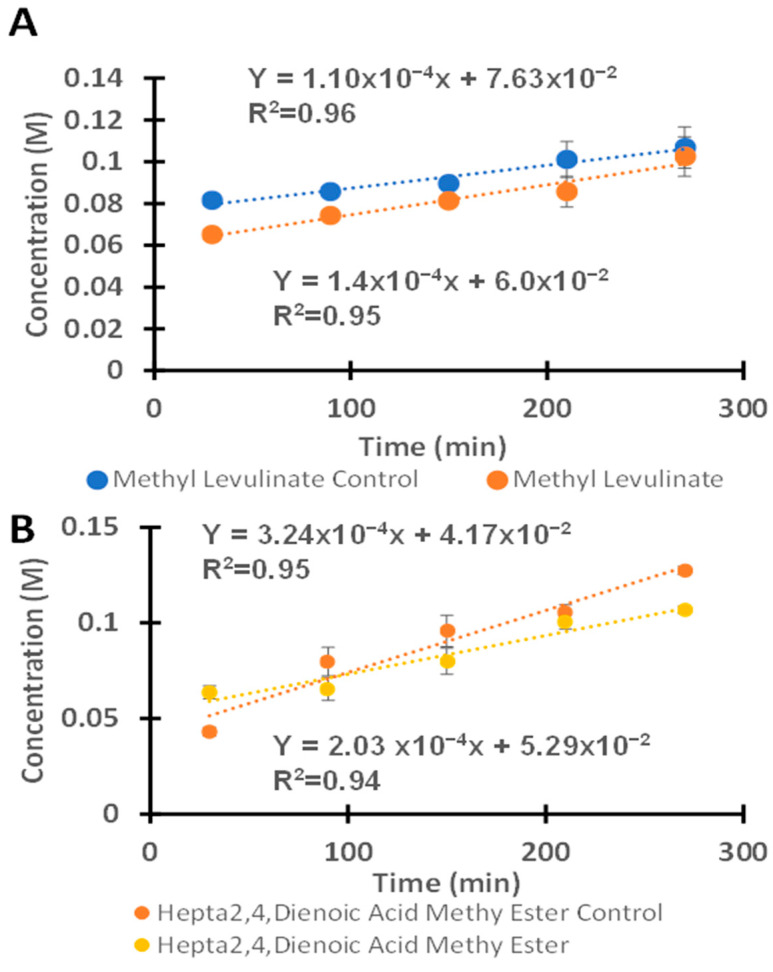
(**A**) The kinetics of the reaction between fructose and HCl with and without the presence of the vanadyl phthalocyanine for the formation of methyl levulinate. (**B**) The reaction of fructose with HCl in the presence of vanadyl phthalocyanine as a catalyst for the formation of hepta-2,4-dienoic acid methyl ester.

As shown in Figure 7A, the reaction performed in the HCl–methanol system presents higher rates of formation of methyl levulinate, with rate constants of 1.1 × 10^−4^ M/min (1.83 × 10^−6^ M/s) and 1.4 × 10^−4^ M/min (2.33 × 10^−6^ M/s) for uncatalyzed and catalyzed reactions, respectively. The catalyzed reactions proceeded approximately 1.3 times faster than the uncatalyzed reactions. The lower catalytic activity observed in H_2_SO_4_–methanol systems catalyzed with VOPC may be attributed to interactions between the catalyst and HSO_4_^−^ ions in solution, hindering access of the fructose to the vanadium ion, thus reducing the catalytic activity. The reaction selectivity toward methyl levulinate in the H_2_SO_4_ system was 69.4 ± 0.23% and 75.5 ± 0.80% for the non-catalyzed and catalyzed reactions, respectively. In the HCl system, although the catalyzed reaction rate was higher in the presence of vanadyl phthalocyanine, the reaction selectivity remained approximately the same, with values of 54.7 ± 1.5% and 50.2 ± 2.4% for the non-catalyzed and catalyzed reactions, respectively. Overall, the selectivity toward methyl levulinate was approximately 20% higher in the H_2_SO_4_–methanol system compared to the HCl–methanol system. Furthermore, the reactions proceeded more selectively in the H_2_SO_4_–methanol system than in the HCl–methanol system. Overall, the reaction performed in the HCl–methanol system was more effective for methyl levulinate production compared to the H_2_SO_4_–methanol system at low temperatures [51,52,53]. Additionally, the formation of the hepta-2,4-dienoic acid methyl ester with the H_2_SO_4_–methanol was also observed to increase with increasing time, as shown in the HCl–methanol in Figure 7B. Changing the acid in the reaction system influenced both the reaction rate and the product selectivity. The H_2_SO_4_ demonstrated higher selectivity towards the methyl levulinate, a highly desirable precursor in the synthesis of food additives, polymers, and synthetic fuels [1,2,3]. The vanadyl phthalocyanine in HCl exhibited higher rate constants for the formation of both methyl levulinate and hepta-2,4-dienoic acid methyl ester, indicating that both the catalyst (VOPC) and acid used significantly affect the reaction selectivity. Typical reaction conditions reported in the literature involve elevated temperatures ranging from 110 to 200 °C and high pressures [54,55]. However, the present reaction system achieved comparable reaction rate constants at atmospheric pressure and a temperature of 64.7 °C.

In the current study, methyl levulinate synthesis yields ranged between 60 and 70%, comparable to or higher than most systems reported in the literature. Hu et al. reported conversions of approximately 20% using C5 carbohydrates in methanol media using Amberlyst 70 and Pd/Al_2_O_3_ catalyst at 165 °C and 20 bar, while achieving approximately 80% yield when starting with 2-furylmethanol under similar conditions [56]. Peng et al. showed that in a methanol medium with low concentrations of H_2_SO_4_, approximately 50 mol% of the initial glucose was converted into methyl levulinate, following a first-order kinetic model [57]. Kuster et al. found that the reaction of fructose in methanol with 3.6-7.2% hydrochloric acid at 90 °C led to a methyl levulinate yield of approximately 81%, independent of the catalyst concentration [58]. Son et al. used Amberlyst-15 as a solid catalyst for the synthesis of levulinic acid from fructose, achieving a 47% isolated yield. The authors reported that the rate of formation of the levulinic acid increased with increasing temperature and was maximized at approximately 135 °C [59]. Thapa et al. used Dowex 50 × 8−100 resin to catalyze the conversion of fructose to levulinic acid in water-based solvents, achieving yields of up to 72 at 120 °C [60]. Weiqi and Shubin reported that glucose conversion into levulinic acid over Cr/HZSM-5 catalyst at 180 °C for 180 min resulted in a yield of approximately 64%, and the reaction kinetics were described by a pseudo-first-order reaction model [61].

## 4. Conclusions

To the best of the author’s knowledge, this is the first time that vanadyl phthalocyanine has been used for the conversion of a sugar (fructose) into value-added products. This study shows the potential development of a low-cost catalytic system with product selectivity, capable of generating feedstock for various potential industrial applications. Vanadyl phthalocyanine was successfully synthesized and evaluated for its catalytic activity in the conversion of fructose to methyl levulinate. X-ray diffraction analysis, followed by Le Bail refinement, revealed the VOPC to be in the P-1 crystal lattice with unit cell parameters a = 12.058 Å, b = 12.598 Å and c = 8.719 Å, and angles of 96.203°, 94.941°, and 68.204°. The reduced χ^2^ of 1.67 indicated a strong agreement between the experimental pattern and the crystallographic data from the literature. FTIR spectroscopy confirmed the metalation of the phthalocyanine ring, as evidenced by the absence of the N–H stretching vibration present in H_2_PC and the shift of the benzene ring deformation coupled with aza deformation from 944 cm^−1^ to 964 cm^−1^ in the VOPC spectrum. Additionally, a peak at 953 cm^−1^ was observed in VOPC, consistent with the V=O stretching vibration. The XPS data supported the presence of the vanadyl environment, its binding with nitrogen, and its effects on the pyridinic and pyrrolic bonds present in the synthesized metal-substituted sample.

The kinetic studies revealed that the reaction followed zeroth order kinetics both in the presence and absence of the vanadyl phthalocyanine. The reactions showed that the reaction rate increased in the presence of the vanadyl phthalocyanine, confirming the catalytic role of the vanadyl phthalocyanine. For the H_2_SO_4_–methanol reaction system with the VOPC, the zeroth order rate constant of 1.10 × 10^−6^ M/s observed was 1.74 times faster than the sulfuric acid alone. In the HCl–methanol system, a higher rate constant of 2.33 × 10^−6^ M/s was observed, which was 1.3 times faster compared to the uncatalyzed reaction in the HCl–methanol. The reaction selectivity toward methyl levulinate in the H_2_SO_4_ system was 69.4 ± 0.23% and 75.5 ± 0.80% for the non-catalyzed and catalyzed reactions, respectively, whereas in the HCl system, reaction selectivity remained approximately the same, with values of 54.7 ± 1.5% and 50.2 ± 2.4% for the non-catalyzed and catalyzed reactions, respectively. The selectivity towards methyl levulinate was approximately 20% higher in the H_2_SO_4_–methanol system compared to the HCl–methanol system.

Comparing both acid systems, methyl levulinate formation was more selective in the H_2_SO_4_–methanol system, though the overall reaction rate was approximately twice as fast in the HCl–methanol system. In all reaction systems, three reaction products were identified; the two major products were methyl levulinate and hepta-2,4-dienoic acid methyl ester, along with a minor product observed in the chromatograms, hydroxymethylfurfural.

The results of the study indicate that vanadyl phthalocyanine may be effectively used as a catalyst to increase the rate of reaction in the production of methyl levulinate, a very important starting material for various chemicals and materials.

## Figures and Tables

**Table 1:** FTIR table for peak position and stretching assignments for the metal-free phthalocyanine, H_2_PC, and the V=O substituted phthalocyanine, VOPC [40,41,42,43,44,45,46,47,48,49].

H_2_PC Peak Position (cm^−1^)	VOPC Peak Position (cm^−1^)	Assignment
710		C-N
729	724	C-H out-of-plane deformation
762	751	Macrocycle ring stretching
778	775	C-N stretching
	801	isoindole stretching coupling + N-V-N asym. stretching
839		C-N-C Ring Breathing
872	876	aza stretching coupling with isoindole deformation
	898	Aza ring shift due to V
944		ring benzene deformation coupling with aza deformation
	953	V=O
	964	benzene deformation coupling with aza deformation
998	1000	Benzene ring and C=C
1064	1064	ν(C–N) stretching in pyrrole vibration
1075	1075	beta (C–H) in plane deformation
1091		
1116	1118	beta (C–H) in plane deformation
1157	1159	ν (C–N) in plane and δ (C–H) in-plane
1187	1192	isoindole stretching
1275		
1299	1284	ν (C–N) in isoindole
1324		
1336	1332	ν (C–C) in isoindole
1417	1418	isoindole stretching
1437		
1461	1461	ν (C–H) in plane bending
1477	1475	ν (C=N) pyrrole
1501	1497	w C–H bending in aryl
1523	1521	beta (C–H) aryl
1576		
1595	1587	benzene C-C stretching
1610	1607	ν (C–C) stretching vibration in pyrrole
2923	2923	ν (C–H) asymmetric stretching
3004	3004	C=C-H
3050	3050	ν (C–H) asymmetric stretching vibration in alkyl
3282		N-H

**Table 2:** Le Bail fitting parameters for the H_2_PC and the VOPC as synthesized.

Compound	Space Group	a (Å)	b (Å)	c (Å)	α (°)	β (°)	γ (°)	χ^2^
α-H_2_PC_synth_	C2/n	25.755	3.773	23.398	90	93.111	90	2.07
α-H_2_PC_lit_	C2/n	26.121	3.797	23.875	90	94.16	90	-
VOPC_synth_	P-1	12.058	12.598	8.719	96.203	94.941	68.204	1.67
VOPC_lit_	P-1	12.027	12.571	8.690	96.04	94.80	68.20	-

## Data Availability

All the relevant data that support the findings of this study are available from the corresponding authors on reasonable request.
